# Role of Repeated Conformational Transitions in Substrate Binding of Adenylate Kinase

**DOI:** 10.1021/acs.jpcb.2c05497

**Published:** 2022-10-12

**Authors:** Jiajun Lu, David Scheerer, Gilad Haran, Wenfei Li, Wei Wang

**Affiliations:** † Department of Physics, National Laboratory of Solid State Microstructure, Nanjing University, Nanjing210093, China; ‡ Wenzhou Key Laboratory of Biophysics, Wenzhou Institute, University of Chinese Academy of Sciences, Wenzhou, Zhejiang325000, China; § Department of Chemical and Biological Physics, Weizmann Institute of Science, Rehovot761001, Israel

## Abstract

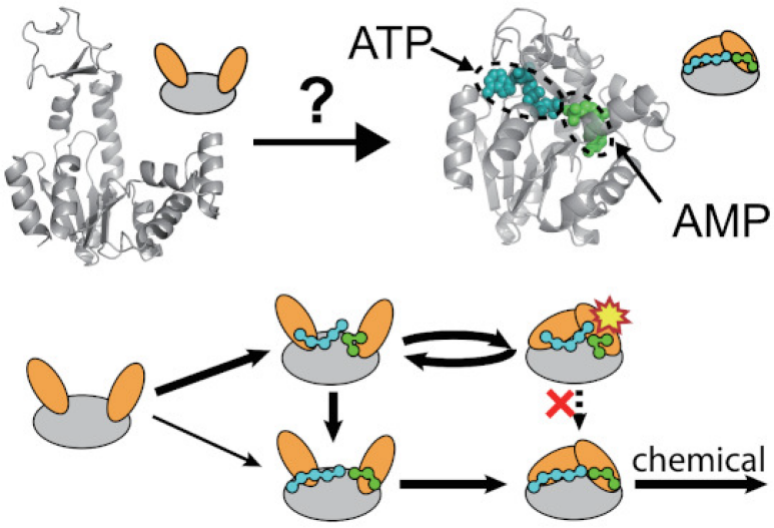

The catalytic cycle of the enzyme adenylate kinase involves large conformational motions between open and closed states. A previous single-molecule experiment showed that substrate binding tends to accelerate both the opening and the closing rates and that a single turnover event often involves multiple rounds of conformational switching. In this work, we showed that the repeated conformational transitions of adenylate kinase are essential for the relaxation of incorrectly bound substrates into the catalytically competent conformation by combining all-atom and coarse-grained molecular simulations. In addition, free energy calculations based on all-atom and coarse-grained models demonstrated that the enzyme with incorrectly bound substrates has much a lower free energy barrier for domain opening compared to that with the correct substrate conformation, which may explain the the acceleration of the domain opening rate by substrate binding. The results of this work provide mechanistic understanding to previous experimental observations and shed light onto the interplay between conformational dynamics and enzyme catalysis.

## Introduction

Natural enzymes have evolved to catalyze chemical reactions in vivo with enormously high efficiency.^1^ Enzymatic catalysis is often accomplished by cycles, including not only chemical reaction steps but also other physical steps, such as ligand exchange and protein conformational motions. One key task in enzyme studies is to address the question of how enzymes coordinate all of these individual steps to achieve high catalytic efficiency.

**Figure 1:**
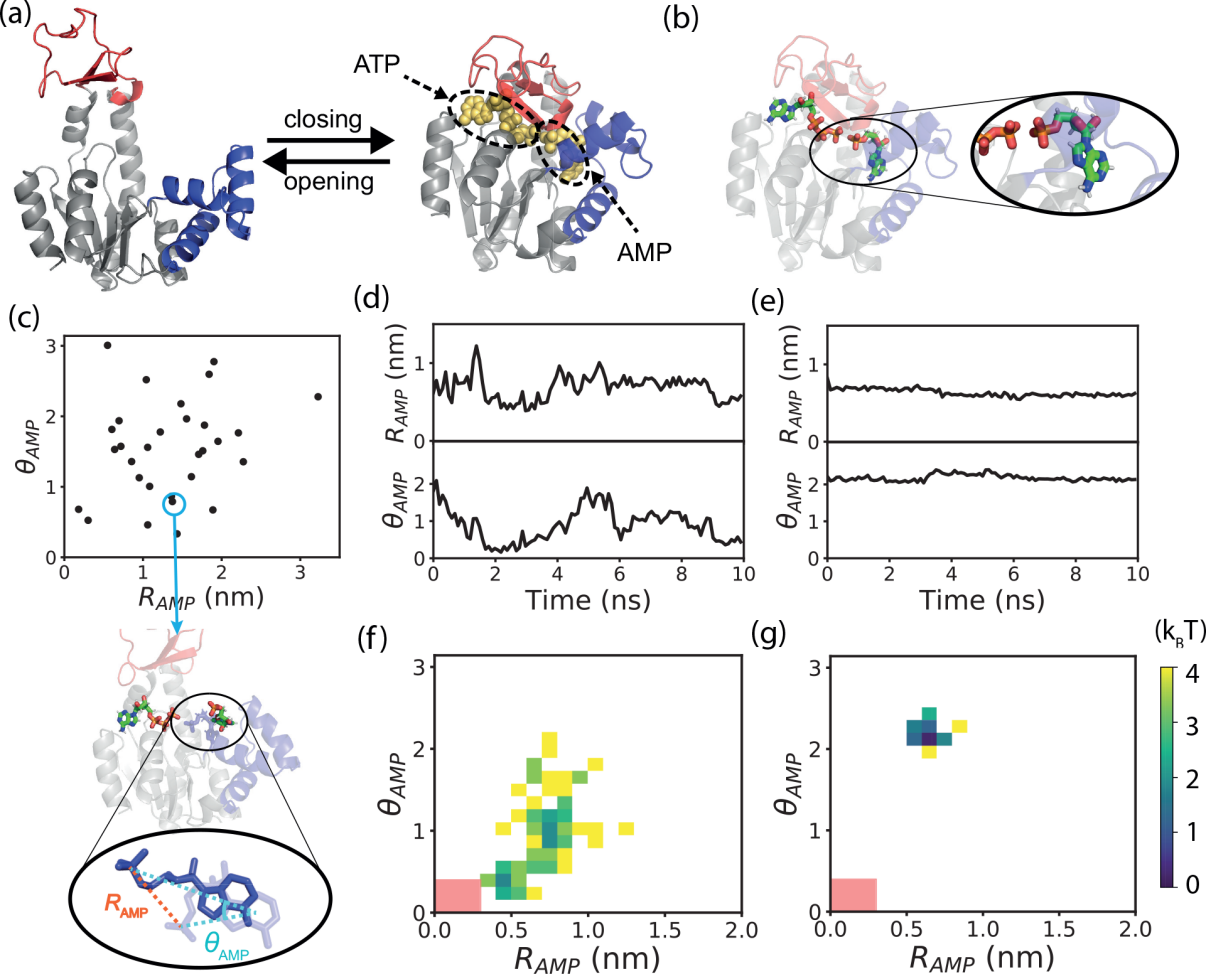
Simulation system and effects of enzyme conformation on the substrate dynamics. (a) Cartoon representations of the three-dimensional structures at the open conformation (left, PDB code: 4AKE) and closed conformation (right, PDB code: 1AKE). (b) Cartoon representation of the AdK structure with correctly bound substrates. Zoom-in panel shows the native binding pose of the substrate AMP by stick representation. (c) Binding poses of the substrate AMP for the encounter complex structures from 30 independent AMP binding simulations. Binding poses of the substrate AMP were projected onto the reaction coordinates θ_*AMP*_ and *R*_*AMP*_ defined in the Methods section. Non-native binding pose for one of the encounter complexes is shown by dark blue in the bottom. For comparison, corresponding native binding pose of AMP is also shown below by light blue. (d and e) Substrate dynamics at the open (d) and closed (e) conformations characterized by the reaction coordinates θ_*AMP*_ and *R*_*AMP*_. (f and g) Free energy landscapes along the reaction coordinates *R*_*AMP*_ and θ_*AMP*_ at the open (f) and closed (g) conformations calculated based on one single trajectory. Area around the native binding pose is marked by a red square.

Adenylate kinase (AdK), which catalyzes the phosphoryl transfer reaction ATP + AMP *⇌* ADP + ADP, has been widely used as a model system to study the interplay between conformational motions and enzymatic catalysis both experimentally and computationally.^2−29^ This enzyme is composed of three domains, i.e., the CORE domain, the LID domain (ATP binding site), and the NMP domain (AMP binding site) (Figure 1a and 1b). The catalytic cycle of AdK involves conformational transitions between a closed conformation and an open conformation. Ligand exchange tends to occur at the open conformation, whereas the chemical reaction requires a closed conformation with the substrates and the residues at the active site being precisely preorganized.^30^ In the canonical models of the AdK catalytic cycle, the substrates first bind the enzyme at the open conformation, which is followed by closure of the LID and NMP domains. After the chemical reaction at the closed state, the enzyme opens its conformation to release the products. Accordingly, one round of domain opening and closing motions is expected for each catalytic turnover event. In addition, early experimental studies showed that substrate binding tends to accelerate the domain closure^4^ but does not affect or even slows down the domain opening.^4,6^ However, a recent experimental work with single-molecule FRET (smFRET) spectroscopy showed that substrate binding increases dramatically both the domain closing and opening steps, and the conformational motions are much faster than the enzyme turnover, suggesting that more than one opening and closing cycle is required for one turnover event.^31^

The unusual effect of the substrate binding on the conformational dynamics of AdK revealed in the above smFRET experiment may suggest that the energy landscape of substrate binding is rugged. As a result, the substrates tend to encounter incorrect binding poses, and repeated cycles of conformational rearrangement are required for them to find the correct binding pose that is competent for chemical reaction.^31^ However, directly demonstrating the role of the repeated conformational cycles for the productive substrate binding is extremely challenging. In a previous computational work,^19^ Matsunaga and co-workers showed that the AMP ribose part of the inhibitor AP_5_A can misbind to the nonspecific site at the CORE domain of AdK, which may prevent the ligand from entering the AMP binding pocket. In addition, there are extensive molecular simulation studies on the effect of substrate binding on the conformational dynamics of AdK.^13,16,22,24,27,28,32,33^ In comparison, a detailed characterization of the role of conformational motions of AdK in productive substrate binding is still rare.

Here, we studied the role of repeated conformational transitions in the substrate binding of AdK by combining all-atom and coarse-grained molecular dynamics simulations. The results directly showed that repeated conformational cycles between the closed state and the open state can lead to a higher probability of successful substrate binding to the correct sites. In addition, by combining the parallel cascade selection molecular dynamics (PaCS-MD) with the umbrella sampling method,^34,35^ we showed that incorrect substrate binding poses highly favor the open conformation of AdK, as they have a much lower free energy barrier for the conformational transition from closed to open states. In comparison, the enzyme with the native substrate binding pose dominantly adopts the closed conformation. Such results suggest that the accelerated domain opening observed in the smFRET experiment may arise from the incorrect binding of the substrates. These results provide a possible molecular mechanism for the experimentally observed effect of substrate binding on the conformational dynamics, therefore shedding light onto the general principles utilized by natural enzymes to achieve enormous catalytic power.

## Methods

### All-Atom MD Simulations

All-atom molecular dynamics simulations were conducted by using Gromacs 2018.8^36^ and Plumed 2.6.2^37^ with the Amber ff14SB force field.^38^ The force field parameters of the AMP were prepared by using the general AMBER force field (GAFF)^39^ and the Antechamber package. The force field parameters of the ATP were taken from Carlson and co-workers.^40^ The protein AdK was solvated in a TIP3P water box with sodium ions added to neutralize the system.^41^ The structures of the open and closed AdK were taken from the protein data bank entries 4AKE(42) and 1AKE,^43^ respectively (Figure 1a). Atomic coordinates of the substrates ATP and AMP were extracted from those of the ligand AP_5_A by removing the δ-phosphate group (Figure 1b). The position of the magnesium ion was taken from PDB entry 1ZIO(44) by superposing the ligand AP_5_A in the two structures.

To investigate how the repeated conformational cycles contribute to the successful substrate binding, we prepared a starting structure with the substrate incorrectly bound to the open conformation of the AdK. For simplicity and without loss of generality, the substrate ATP was bound with the native pose while AMP was bound with a non-native pose. The initial atomic coordinates for the non-native pose of the AMP were prepared by performing a rigid body rotation of the AMP molecule around the C1′–N9 bond by 180° from the native binding pose. On the basis of the above ligand coordinates at the closed protein conformation, we then constructed the atomic coordinates for the open conformation of AdK with a non-native ligand binding pose by superposing the atoms of the CORE domain in the open and closed structures of the protein.

The simulation system was first minimized for 10 000 steps. Then, the system was heated to 300 K within 50 ps in the NVT ensemble. The system was further relaxed for another 300 ps in the NPT ensemble at 300 K and 1.0 atm. The relaxed structures were then used as the starting structures for the subsequent production simulations. The molecular structures were visualized by the software PyMOL.^45^

### PaCS-MD Simulations of the Conformational Transitions

As the time scale involved in substrate binding and conformational motions of AdK is far beyond the capability of conventional all-atom molecular dynamics, we used parallel cascade selection molecular dynamics (PaCS-MD) to simulate the repeated opening/closing transitions of AdK. PaCS-MD uses a genetic-like algorithm to guide the conformational sampling toward a known target structure.^34^ During the PaCS-MD simulations, multiple short trajectories (0.1 ns) were run in parallel, and the snapshots closest to the target structure were selected from the structural ensemble sampled by the short trajectories. These selected structures were then used as the starting structures for the next round of short MD simulations. By repeating the short MD simulation rounds (which were termed PaCS-MD cycles), we were able to sample the conformational transition events between the open and the closed conformations without introducing a biasing potential. The all-atom root-mean-square deviation (*RMSD*) with respect to the target structure was used as the reaction coordinate to guide the conformational sampling. To improve sampling efficiency, the simulation system was relaxed for 0.1 ns by conventional MD simulations if PaCS-MD could not find any conformation closer to the target structure.

We also conducted simulations with the protein maintained at the closed conformation but with a non-native ligand binding pose. To avoid steric conflicts, we first used the PaCS-MD simulations starting from the open conformation to sample the closed conformation, during which a positional fix was applied to the substrates. Then we performed target MD simulations with the closed conformation used as a reference to maintain the protein at the closed conformation by applying a harmonic restraint on the heavy protein atoms along the chosen reaction coordinate. The *RMSD* with respect to the closed conformation was used as the reaction coordinate in the target MD simulations, and the force constant of the harmonic restraint was set to 23 900 kcal/mol/nm^2^.

In the PaCS-MD simulations and the conventional MD simulations at the closed and open conformations, a restraint potential was applied to the substrate ATP, so that it always stayed at the corresponding binding site with the native pose. We also imposed a weak biasing potential toward the native pose for the heavy atoms in the purine ring of the substrate AMP in order to enhance the sampling of the productive binding event. The restraint potential was given by , where *H* is the Heaviside step function, *r* represents the substrate *RMSD* with respect to the native position, and *r*_0_ is the corresponding reference value of *RMSD*. In calculating the *RMSD* values, the residues of AdK at the binding sites were aligned to those at the PDB structure, and the heavy atoms in the purine ring of the substrate AMP were used in the calculation. The remaining part of the substrate AMP was allowed to move freely. The parameters *K* and *r*_0_ were set as 23.9 kcal/mol/nm^2^ and 0.2 nm, respectively. With such a weak biasing potential to the substrate AMP, one can successfully sample the productive AMP binding events within a reasonable simulation time using the PaCS-MD simulations but simultaneously allowing the substrate AMP to relax flexibly around the binding site.

### Reaction Coordinates Characterizing the Substrate AMP Binding

Two quantities, *R*_*AMP*_ and θ_*AMP*_, were defined to characterize the correctness of AMP binding (Figure 1c). In a given MD snapshot (denoted by *X*), the CORE domain of AdK was superimposed to the crystal structure in the closed conformation (denoted by *C*). *R*_*AMP*_ was defined as the distance between the terminal phosphorus atom of the substrate AMP in conformations *X* and *C*. The center of mass of the base and the terminal phosphorus atom of AMP were connected to obtain a vector, and then, θ_*AMP*_ was defined as the angle formed by the above-defined vector in each of these conformations (Figure 1c). The values of *R*_*AMP*_ and θ_*AMP*_ were expected be close to 0 when the AMP conformation was in the native pose. Similar definitions of *R* and θ were applied to ATP. The substrate binding was considered correct if the values of the two reaction coordinates satisfied the conditions *R* < 0.3 nm and θ < 0.4 for a certain time interval when AdK was at the closed conformation.

As an alternative, we also used the reaction coordinate *Q* to characterize the correctness of substrate binding, which was defined as the fraction of formed native atomic contacts between the substrate and the protein. The native contacts were defined as heavy atom pairs from the ligand and protein with a distance less than 0.35 nm. *Q* was defined as , where *N* is the number of native contacts and the sum is over the native contact pairs. In this work, substrate binding was considered correct if *Q* ≥ 0.4, a relatively loose criterion.

### Umbrella Sampling

The free energy landscapes of the conformational transition of AdK were constructed based on umbrella sampling simulations.^35^ We performed two sets of umbrella simulations with the native and non-native ligand binding poses, respectively. The reaction coordinate used in the umbrella sampling simulations was defined as
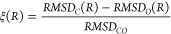
1where *RMSD*_*C*_(*R*) and *RMSD*_*O*_(*R*) represent the all-atom root-mean-square deviations of a given structure *R* with respect to the closed and open structures, respectively, while *RMSD*_*CO*_ represents the root-mean-square deviation between the closed and the open structures. By such a definition, values of −1 and 1 of the reaction coordinate ξ(*R*) correspond to the closed and open structures, respectively. The whole range of the reaction coordinate ξ(*R*) was divided into 41 windows with an interval of 0.05. The force constant of the umbrella sampling was set as 836 kcal/mol. The initial structures of the umbrella sampling were prepared based on the above PaCS-MD simulations. For the umbrella windows with ξ < – 0.1 (ξ > 0.1), the initial structures were taken from the closed to the open (open to closed) trajectories of the PaCS-MD simulations. In contrast, for the umbrella windows with −0.1 ≤ ξ ≤ 0.1, we performed the simulations with two sets of initial structures, which were taken from the PaCS-MD trajectories along the two opposite directions. The simulation length of each window was set as 40 ns, and the snapshots of the first 10 ns in each window were discarded in the analysis. The MBAR method was used to reweigh the sampled structures in different windows and construct the free energy landscapes of the conformational transition.^46^

### Coarse-Grained Molecular Simulations

Although the PaCS-MD simulations allow the sampling of repeated conformational transitions, it is still extremely challenging for the substrate to sample a wide range of binding conformations. To achieve a more quantitative characterization of the role of conformational motions on substrate binding, we constructed a coarse-grained model to simulate the coupled substrate binding and conformational motions of AdK. Similar coarse-grained models have been widely used in the modeling of ligand binding coupled protein allosteric motions.^25,27,28,47−49^

In the coarse-grained model of AdK, each amino acid was represented by one spherical bead located at the Cα position. The multiple-basin energy function combined with the atomic interaction-based coarse-grained model (AICG2+) were used to simulate the conformational motions of the AdK.^50,51^ Following previous work,^52^ the energy function of the enzyme at the apo state is given by . Here, the summation index *i* runs over three components describing the LID-CORE interactions, NMP-CORE interactions, and LID-NMP interactions. The component  has a double-basin topology dictating the conformational switching of the enzyme between the closed and the open conformations and is given as follows^50^
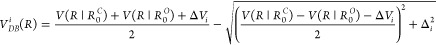
2In the above double-basin energy function, the terms  and  are the structure-based AICG2+ potentials centric to the closed and open structures, respectively, which are given by . Here, *R* and *R*_0_ collectively represent the coordinates of the coarse-grained beads in a given protein structure and the reference PDB structure, respectively. The reference structures at the closed (open) conformations were taken from the protein data bank with the entry 1AKE (4AKE). *V*_*bond*_ represents the covalent interactions between the consecutive beads. *V*_*loc*_^*FLP*^ is the flexible local potential, which was extracted by statistical survey of the coiled library.^53,54^*V*^*SB*^ is the structure-based potential term shaping up a funneled energy landscape that drives the folding of the protein to its native structure.^55−57^*V*_*exv*_ represents the excluded volume term. The energy gap parameters Δ*V*_*i*_ in the double-basin energy function were taken from previous work, with which the experimentally measured conformational equilibrium (i.e., the population of the closed conformation is ∼0.3 at the apo state) can be reproduced.^52,58^ Δ is the coupling parameter, which was set such that the conformational transition occurs within a reasonable simulation time. The resulting model corresponds to the wild-type enzyme and is referred to as the WT model in this work. As a control, we also constructed a modified model, in which the energy gap parameters Δ*V*_*i*_ and the coupling parameters Δ_*i*_ were tuned such that the enzyme dominantly stayed in the closed conformation at the apo state, and the resulting model is referred to as the C model.

The substrate molecules ATP and AMP were represented by seven beads and five beads, respectively. The interactions between the protein and the substrates were described by the following energy function
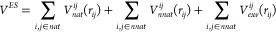
3In the above energy function, the terms *V*_*nat*_^*ij*^, *V*_*nnat*_^*ij*^, and *V*_*exv*_^*ij*^ represent native interactions, non-native interactions, and excluded volume interactions between the residue bead *i* and the substrate bead *j*, respectively. The native interaction applies to native contacts between the substrate and AdK and is given by
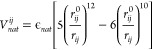
4where *r*_*ij*_ is the distance between the AdK bead *i* and the substrate bead *j* and *r*_*ij*_^0^ is the corresponding reference value given in the PDB structure. The distance threshold for defining a native contact is 6.5 Å between the heavy atoms of the substrate and protein. The parameter ε_*nat*_ = 0.15 kcal/mol describes the strength of the protein–substrate interactions. *V*_*nat*_^*ij*^ provides a minimally frustrated energy surface for substrate binding. To describe the ruggedness of the substrate binding landscape, we introduced the non-native term *V*_*nnat*_^*ij*^ for substrate–protein residue pairs not forming native contacts. Only protein residues within 6 Å from the residues forming native contacts were considered. Meanwhile, the distances of the non-native residue pairs at the native structure were not allowed to be larger than 10 Å to avoid overlap between native and non-native interactions. The energy function of the non-native interactions was given by
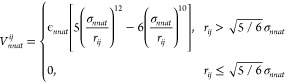
5The parameter σ_*nnat*_ was set as 6.0 Å. The strength of the non-native interaction ε_*nnat*_ controls the ruggedness of the substrate binding energy landscape. In this work, a wide range of values of ε_*nnat*_ were used to investigate the effect of energy landscape ruggedness on the substrate binding dynamics. We also applied an excluded volume term  for the residue pairs between the substrate and the protein molecules without forming native contacts (with σ = 5.0 Å) and between the ATP and the AMP molecules (σ = 3.5 Å). The coefficient ε_*exv*_ was set as 1.0 kcal/mol. For simplicity, ATP and AMP were treated as rigid-like ligands in which the bonds, angles, and dihedral angles formed by the coarse-grained beads were restrained to their reference values corresponding to those in the PDB structure using a structure-based energy function.^59^

To characterize the coupling between substrate binding and conformational motions, we defined the following reaction coordinates *R*_*LID*–*CORE*_, *R*_*NMP*–*CORE*_, *Q*_*ATP*_, and *Q*_*AMP*_. Here, *R*_*LID*–*CORE*_ (*R*_*NMP*–*CORE*_) represents the distances between the centers of mass of the CORE domain and the LID (NMP) domain. *Q*_*ATP*_ (*Q*_*AMP*_) represents the fraction of the formed native contacts between the ATP (AMP) and the protein, which was defined as . Here, *N* is the number of native contacts between the substrate and the enzyme, and the sum is over the native contact pairs. The substrates were considered as correctly bound when *Q*_*ATP*_ ≥ 0.7 and *Q*_*AMP*_ ≥ 0.7.

All of the coarse-grained simulations were performed by a modified version of the CafeMol2.0 software.^60^ The temperature of the simulations was controlled by a Langevin thermostat with the friction coefficient set to γ = 0.25 τ^–1^ and the temperature at *T* = 300 K. The time step was set as 0.1 τ, with τ being the reduced time unit in CafeMol.

To quantify the rates of ligand binding, we calculated the mean first passage time (MFPT) for the successful ligand binding by performing 96 independent simulations. The simulations were started from unbound structures with the substrate molecules randomly positioned under the WT model. In the C model, the simulations were started from the snapshots of the first closing event of AdK in the WT model simulations. The trajectories with the substrate molecules staying out of the binding area in the initial structure were excluded in calculating the MFPT for the C model and in the computation of free energy profiles of the WT model. Distance restraints were applied to the substrates to restrain them inside the binding area. We also calculated the MFPT for the opening of the LID and NMP domains of the WT model without substrate binding, with substrate bound correctly, and with substrate bound incorrectly. The initial structures for the simulations with substrate bound were extracted from the above-mentioned binding simulations. The closed structures with *Q* ≥ 0.7 and *Q* < 0.5 were selected as the initial structures of the simulations for the substrate correctly bound state and incorrectly bound state, respectively. To ensure that all conformational opening simulations were started from the closed conformation, these initial structures were first relaxed for 2 × 10^5^ MD steps at the closed state by using the C model. MFPTs were then calculated by a maximum likelihood estimation.

## Results

### Structural Heterogeneity of the Substrate–Enzyme Encounter Complex

We first investigated the initial stage of the substrate binding events. Sampling the large-scale motions of substrates around the binding sites by all-atom MD simulations is very challenging. For simplicity, the simulations were initiated from the open conformation with ATP being correctly prebound at the corresponding binding site and AMP being positioned outside the binding area. Meanwhile, a distance restraint (distance between the centers of mass of the AMP and the binding site residues) was applied to the substrate AMP, so that it could not fly far away from the binding site and one could sample binding events within reasonable simulation time. We performed 30 independent binding simulations with a length of 140 ns, and the final snapshots of the trajectories were considered as the encounter complexes for analysis. Figure 1c shows the structural features of the encounter complexes projected onto the reaction coordinates θ_*AMP*_ and *R*_*AMP*_. θ_*AMP*_ and *R*_*AMP*_ were used to characterize the deviations of the orientation and distance of the substrate AMP, respectively, from those of the correct binding pose (see Figure 1c and Methods for their definitions). One can see that the initial encounter complexes from different simulation trajectories can adopt diverse structures. Most of these structures have large differences from the native binding structure. Such results suggest that the substrate binding energy landscape is rugged and a large rearrangement is needed for the substrate AMP in the encounter complexes to find the native binding pose.

### Substrate Dynamics at the Open and Closed Conformations of the Enzyme

We then performed MD simulations to investigate the motions of the substrate at the open and closed conformations. In all of these simulations, the substrate ATP was prebound with the native pose for the sake of simplicity whereas the AMP was placed at the binding site with a non-native pose. For the simulations at the closed conformation, the initial closed structure with a non-native AMP binding pose was prepared following the steps described in the Methods section. As discussed later in this work, a substrate with a non-native binding pose tends to destabilize the closed conformation and one can observe a partial domain opening event within a short time interval. For better control, a harmonic restraint potential along the reaction coordinate *RMSD* with respect to the crystal structure of the closed conformation (PDB code 1AKE) was applied to keep the enzyme at the fully closed conformation. For the simulations at the open conformation, we did not apply any restraint to the enzyme conformations.

The exemplary trajectories show that AMP can move freely in the binding pocket when AdK stays at the open conformation, as demonstrated by the large fluctuations of the values of the *R*_*AMP*_ and θ_*AMP*_ (Figure 1d). Not surprisingly, the conformational fluctuation is much suppressed at the closed conformation (Figure 1e). The free energy landscape constructed based on one single trajectory also demonstrates that AMP can access a much wider conformational space at the open conformation than that at the closed conformation (Figure 1f and 1g). As a result, if the substrate binds at the pocket with a non-native pose, it is unlikely to be relaxed to the native pose at the closed conformation without domain opening. These results may suggest that repeated opening and closing transitions are needed for the productive binding of the substrate molecules. The difference of the substrate mobility at the closed and open conformations of AdK was also observed in a previous work based on the molecular simulations with the string method.^19^

### Role of Repeated Domain Opening and Closing on the Productive Substrate Binding

Next, we studied substrate dynamics when AdK can repeatedly close and open, which were simulated by employing PaCS-MD (see Methods for details).^34^ For each round of closing and opening simulations, we first performed conventional MD simulations with a length of 10 ns at the open conformation, which was followed by 200 PaCS-MD cycles to sample the open-to-closed transition event. Then, another 100 PaCS-MD cycles were performed to simulate the closed-to-open transition. We repeated three rounds of closing and opening simulations for each PaCS-MD trajectory. In total, 60 independent PaCS-MD simulation trajectories were collected.

**Figure 2:**
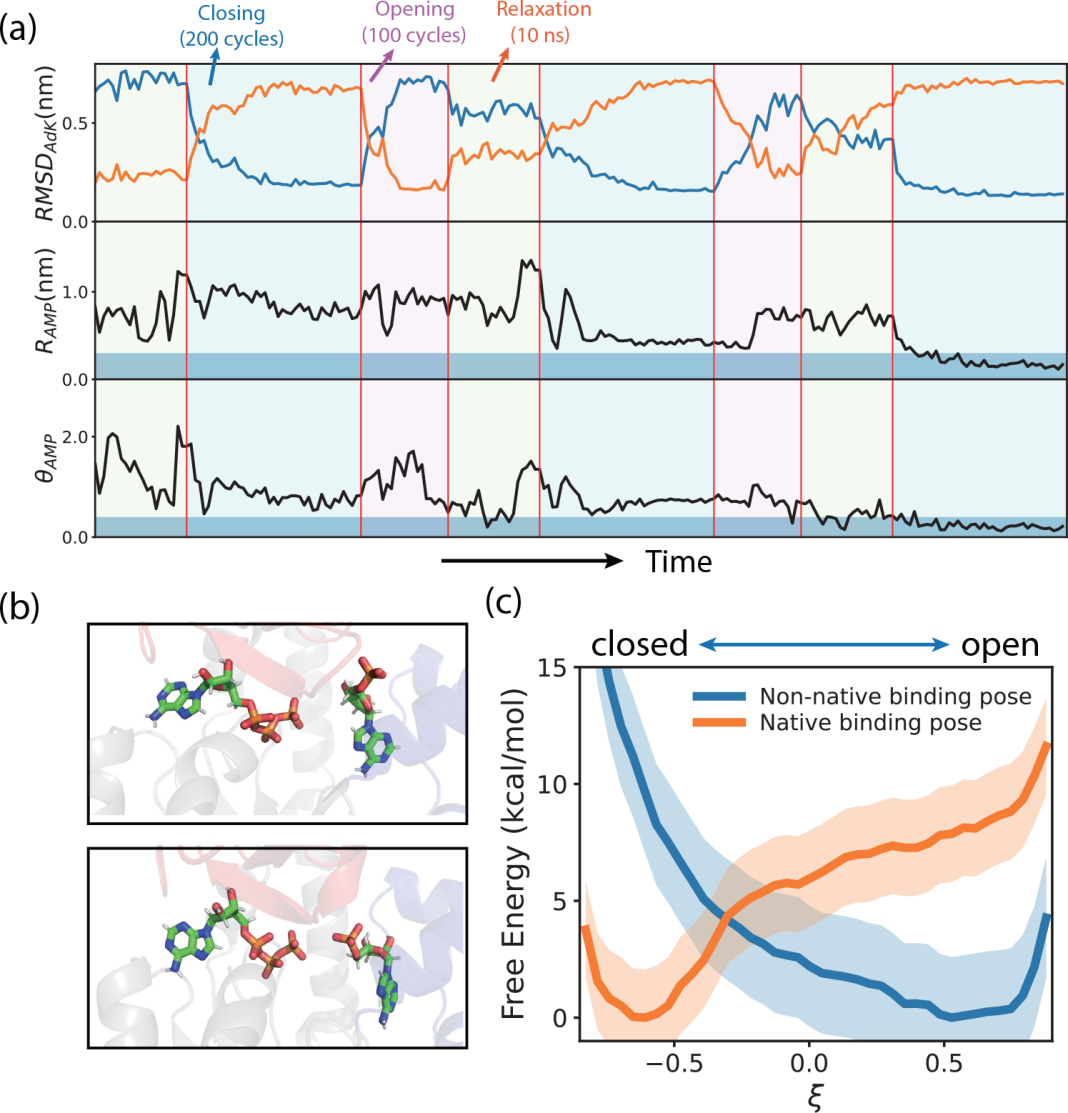
Conformational motions of enzyme facilitate substrate binding. (a) Representative PaCS-MD trajectory showing the coupling between the substrate binding and the conformational transitions. In the top panel, *RMSD*_*AdK*_ represents the root-mean-square deviations of the protein AdK with respect to the closed conformation (blue) or open conformation (orange). In the middle and bottom panels, time series of the reaction coordinates θ_*AMP*_ and *R*_*AMP*_ were plotted to monitor the substrate dynamics. After three rounds of opening and closing conformational transitions, the AMP successfully rearranged to the native binding pose as indicated by the thresholds defined in the Methods section (blue shadowed areas). (b) Three-dimensional structures showing the substrate poses after one round (upper) and three rounds (lower) of opening and closing cycles of PaCS-MD simulations. (c) Free energy profiles along the reaction coordinate ξ. Reaction coordinates ξ with values of −1 and 1 correspond to the closed and open structures, respectively. Error shown by shadows represents the difference of the calculated free energies from two sets of umbrella sampling simulations (see Methods).

Figure 2a shows a representative PaCS-MD trajectory. Note that the open-to-closed transition occurs within the first few tens of PaCS-MD cycles. Therefore, during the remaining part of the 200 PaCS-MD cycles, the enzyme keeps staying around the closed conformation. One can see that the range of conformational space sampled by the substrate AMP is much smaller at the closed conformation than that at the open conformation, which is consistent with the above observations given in Figure 1d–g. For this representative trajectory, after three rounds of closing and opening transitions, the AMP, which initially bound to the enzyme with the non-native pose, successfully relaxed to the native-like binding pose according to the criteria defined in the Methods (Figure 2b). In order to characterize to what degree the two domains are open and closed during the conformational transitions in the PaCS-MD simulations, we calculated the distance between the LID (NMP) domain and the CORE domain for the PaCS-MD trajectory (Figure S1). The results show that the interdomain distances can vary in a wide range between the open and the closed conformations, suggesting that the conformational transitions observed in the PaCS-MD simulations reflect the large-scale closing/opening transitions measured in the single-molecule experiment.^31^

We also plotted all of the trajectories along the interdomain distances *R_LID–CORE_* and *R_NMP–CORE_* for the open-to-closed transitions and the closed-to-open transitions (Figure S2). Consistent with the above discussion, we observe large-scale motions of the LID and NMP domains for all of the PaCS-MD trajectories. Although the transition pathways are highly diverse, we can observe some obvious features of the conformational transitions. Overall, as the ATP is prebound with a native pose in the PaCS-MD simulations, the LID domain can easily access the fully closed conformation during the open-to-closed transitions (Figure S2a). In comparison, it is more difficult for the NMP domain to arrive at the fully closed state because of the non-native binding of the substrate AMP. For the closed-to-open transitions, the trajectories mostly follow the diagonal line in Figure S2b, demonstrating cooperative opening transitions of the two domains. There are some trajectories in which the NMP domain opens later than the LID domain, largely due to the successful relaxation of the substrate AMP to the native binding pose (Figure S3).

As a control, we also performed simulations with the enzyme being maintained at the closed conformation without repeated conformational transition by applying a conformational restraint (see previous subsection). The lengths of the simulations at the closed conformation were 110 ns, which corresponds to the cumulative simulation length of the PaCS-MD trajectories. In total, 20 independent simulations were performed.

In order to characterize the role of conformational transitions in substrate binding, we analyzed the ratio of successful binding events based on the simulations with and without repeated domain opening and closing (Table 1). One can see that during the simulations at the closed conformation with a total length of 110 ns, none of the trajectories can sample the successful substrate binding event if the initial binding pose is incorrect. This result is not surprising because the substrate confined in the binding pocket at the closed conformation has low mobility and therefore cannot relax to the native binding pose. In comparison, after three rounds of repeated open–closed simulations, the substrate AMP can successfully relax into the native-like binding poses for more than one-third of the 60 trajectories, starting from non-native binding pose. It is worth noting that the obtained ratio of successful substrate binding events based on the above PaCS-MD simulations should be much higher than the realistic ratio, because a biasing potential was applied to the substrate AMP to speed up the productive binding for both the PaCS-MD simulations and the relaxation simulations at the closed conformation.

**Table 1:** Ratios of Successful Substrate Binding Events with Different Simulation Schemes and Different Criteria for Substrate Binding

simulation scheme	successful ratio (*R*, θ criteria)	successful ratio (*Q*-score criteria)
open–closed cycle	24/60	23/60
closed	0/20	0/20

Apparently, the improved efficiency of successful AMP binding in the above simulations with repeated conformational transitions is not due to the enhanced sampling implied by PaCS-MD, since the enhancement of the sampling related to PaCS-MD is applied to the conformational motions of AdK, not to substrate binding. Therefore, the above results suggest that the repeated opening/closing transitions can assist the substrate to find the native binding pose, which is consistent with the recent single-molecule experimental observations.^31^

It is worth mentioning that the time scale of the substrate relaxation to the native binding pose depends on the initial encounter structures of the substrate binding. As shown in Figure 1c, the initial encounter structures of the AMP binding are highly diverse. Therefore, the time scale for successful AMP binding would be diverse. When the initial binding pose of the substrate AMP has a small deviation from the native binding pose, domain opening with moderate amplitude would be enough for the substrate reorientation, and it is possible for the substrate to find the native binding pose even without repeated opening/closing transitions. However, when the initial encounter structure has a large deviation from the native binding pose, large-scale adjustment of the substrate is needed. In this case, multiple rounds of domain opening with large amplitude would be essential for the productive substrate binding.

### Incorrect Substrate Binding Facilitates Domain Opening of AdK

To investigate the effect of substrate binding states on the conformational dynamics, we also calculated the free energy landscape of conformational motions by conducting umbrella sampling simulations (see Methods). Figure 2c shows the free energy profiles along the reaction coordinate ξ, which ranges between −1.0 (fully closed conformation) and 1.0 (fully open conformation). One can see that when both ATP and AMP were bound to the binding site with a native binding pose, the closed conformation is much more stable (lower free energy) than the open conformation (higher free energy). These results are consistent with previous computational and experimental studies, which showed that substrate binding stabilizes the closed conformation.^13,22,24,33^ Intriguingly, when the substrate AMP is bound with a non-native binding pose, the closed state becomes much destabilized and domain opening becomes a downhill process along the free energy profile. A previous work by Wolf-Watz and co-workers showed that a GTP molecule can bind to the LID domain site in an inverted pose compared to the native ATP binding pose. They found that AdK does not close due to the non-native pose of GTP, which may be consistent with the above observed destabilization of the closed state by incorrect substrate binding.^61^

The above results may have significant biological implications. When the substrate molecules successfully find the native binding pose, the enzyme will be stabilized at the catalytically competent closed state, therefore favoring the subsequent chemical reaction step. However, when the substrate is bound incorrectly, the resulting downhill-like free energy landscape tends to drive rapid conformational opening, which is required for the substrate rearrangement to find the native-like binding pose as discussed above. These results also suggest that substrate binding may speed up domain opening if the substrate was bound with a non-native pose, which is consistent with the recent experimental observation based on smFRET.^31^

### Role of Repeated Domain Opening and Closing Transitions on Substrate Binding Probed with Coarse-Grained Simulations

**Figure 3:**
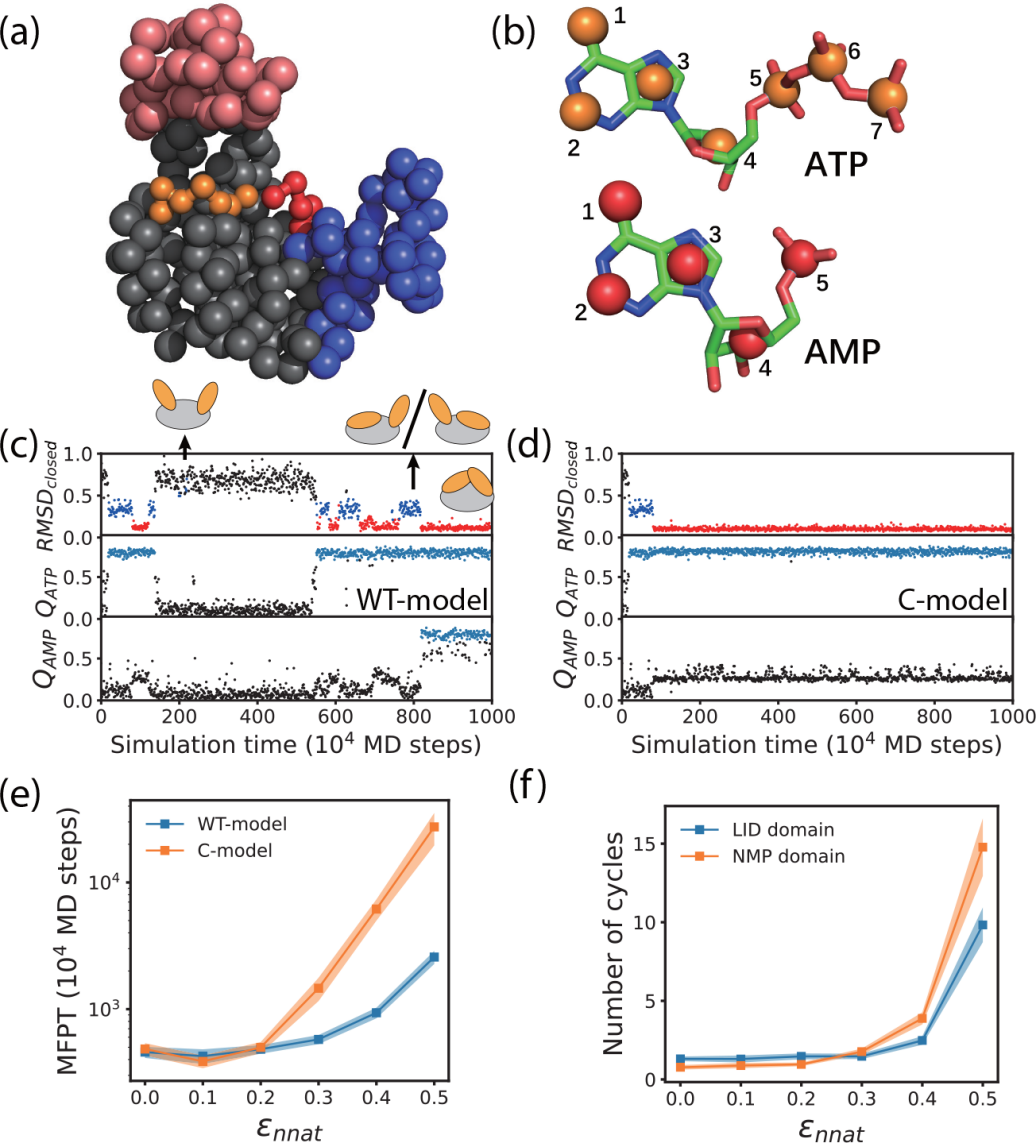
Coarse-grained simulations of substrate binding and conformational motions. (a) Cartoon representation of the coarse-grained structure of the enzyme and substrates. (b) Coarse-grained scheme of the substrates. (c) Representative trajectory showing the time series of the root-mean-square deviation of the enzyme with respect to the closed conformation (*RMSD*_*closed*_) and the fraction of native contacts formed by ATP (*Q*_*ATP*_) and AMP (*Q*_*AMP*_) based on the WT model and with ε_*nnat*_ = 0.5. In the *RMSD*_*closed*_ trajectory (top), red, blue, and black dots represent the fully closed conformation, one-domain closed conformation, and open conformation, respectively. In the *Q*_*ATP*_ and *Q*_*AMP*_ trajectories (middle and bottom), snapshots with successful substrate binding were marked light blue. (d) Representative trajectory based on the C model and with ε_*nnat*_ = 0.5. (e) MFPTs for the substrates to find the native binding pose as a function of the strength of non-native contacting interactions ε_*nnat*_ based on the WT model (blue) and C model (orange). (f) Number of opening and closing cycles of the LID domain (blue) and NMP domain (orange) required for both of the substrates to find the native binding poses as a function of the strength of non-native contacting interactions ε_*nnat*_ based on the WT model. Results are the average of 96 independent trajectories. Error bars were computed by the bootstrap method.

In the above all-atom MD simulations with PaCS-MD, ATP was prebound at the correct position in order to reduce the computational complexity. Therefore, we mainly focused on the coupling between the AMP binding and the domain motions. However, the motions of the LID domain are more strongly coupled to ATP binding. In order to simulate the interplay between the conformational motions and the binding of both substrate molecules, we also constructed a coarse-grained model (Figure 3a and 3b). Similar to the setup of all-atom simulations, an open conformation was adopted for AdK at the initial structures of the simulations. The substrates ATP and AMP were placed randomly around the binding pockets. We first conducted simulations with the wild-type model (WT model) for which AdK was allowed to switch between the open and the closed conformations freely during the simulations (Methods; Figure 3c and Figure S4 in the Supporting Information). On the basis of the simulated trajectories, we calculated the mean first passage time (MFPT) for the substrates to find the native binding pose, which was used to quantify the rate of successful substrate binding. As a control, we also conducted simulations with a control model of AdK (C model) in which the AdK was restrained at the closed conformation once arriving at it without switching back to the open conformation (Methods; Figure 3d and Figure S4 in the Supporting Information).

We calculated the MFPT for successful substrate binding for the WT model and the C model of AdK as a function of the relative strength of non-native contacts ε_*nnat*_ between the substrate and the protein, which controls the ruggedness of the substrate binding energy landscape. With small ε_*nnat*_ values (0–0.2), the frustration of substrate binding is weak and the substrates bind correctly upon the first domain closure event of AdK (Figure 3e). The resulting MFPT values are small and comparable for the WT model and the C model (Figure S4 in the Supporting Information and Figure 3e). Such results suggest that when the energy landscape of the substrate binding is minimally frustrated, the substrate molecules can quickly find the correct binding pose even without repeated conformational switching of the enzyme. When the ε_*nnat*_ value becomes larger (≥0.3), so that the energy landscape is more rugged, the MFPT for successful substrate binding events increases with increasing ε_*nnat*_. The MFPT for the WT model of AdK is significantly smaller than that for the C model of AdK (Figure 3c–e). Correspondingly, a larger number of repeated domain opening and closing cycles is needed for the successful binding of both substrates. The number of conformation cycles increases with increased strength of non-native contacts for the WT model (Figure 3f). Such results clearly demonstrate that repeated conformational switching can facilitate productive substrate binding events. These results are consistent with the above all-atom MD simulations. Compared to the NMP domain, the LID domain involves a smaller number of conformational cycles. One possible reason is that the LID domain cannot easily access the closed conformation when the ATP adopts a largely incorrect binding pose. It is worth mentioning that the ruggedness of the substrate binding energy landscape in the above coarse-grained model is described by the parameter ε_*nnat*_. Although the simulation results clearly demonstrate that a larger number of conformational cycles is needed for the substrates to arrive at the native binding poses when the binding energy landscape is more rugged, it is difficult to directly estimate the realistic value of ε_*nnat*_. A single-molecule experiment showed that one turnover event involves a much larger number of conformational cycles,^31^ which may suggest that the ruggedness of the substrate binding energy landscape used in the current coarse-grained model is weaker than the realistic case, allowing to observe a successful substrate binding event within a reasonable simulation time.

**Figure 4:**
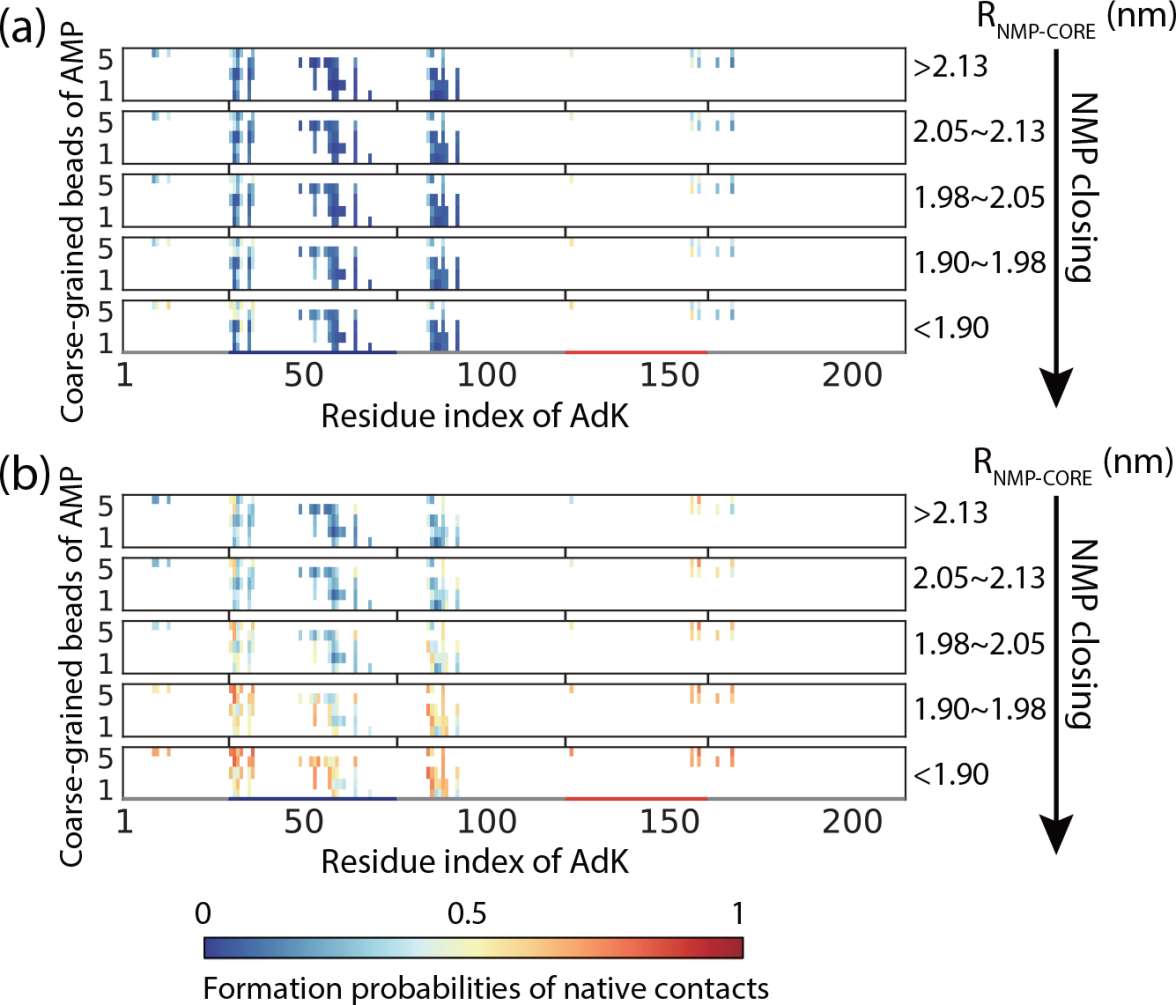
Probabilities of formed native contacts between coarse-grained beads of substrate AMP and protein AdK at different stages of the first open-to-closed transition event (a) and the last open-to-closed transition event (b) of a complete substrate binding trajectory leading to the native binding pose. Results were calculated based on 96 independent simulation trajectories with ε_*nnat*_ = 0.5. For better comparison, trajectories with substrates staying out of the binding area during the conformational closing events were excluded.

There are two possible effects which may contribute to the key role of repeated domain opening/closing motions in productive substrate binding. First, the incorrectly bound substrate has a chance to relax to the native-like pose when the domains are open to some extent as discussed above. Second, the closing motions of the LID and NMP domains may directly push the substrates to the correct poses. To characterize the above two effects, we collected two segments from the substrate binding trajectories leading to the final native binding state. These segments corresponded to the first and last open-to-closed transition event within each trajectory. Then, we calculated the probabilities for the formation of native contacts between the coarse-grained beads of the substrates and the residues in the binding sites during the two segments. Here, we mainly focused on the NMP domain site. The initial position of the substrate AMP is far from the native pose for the first trajectory segment as shown by the low formation probabilities of native contacts at the early stage of the domain closing event (Figure 4a). In contrast, at the beginning of the last trajectory segment, AMP tends to adopt a more native-like pose (Figure 4b). One can see that the closing motions of the NMP domain has a negligible effect on the formation of native contacts during the first open-to-closed transition event. Even after fully closing the NMP domain, the formation probabilities of the native contacts are very low. In contrast, the closing motion of the NMP domain has a significant effect on the formation of native contacts during the last open-to-closed transition. These results suggest that the domain closing action can contribute to the correct substrate binding only when the substrate has already relaxed to a near-native binding pose by repeated conformational switching. Similar results can be observed for the LID domain site, except that LID domain closing events most often only occur when the ATP already adopts a near-native binding pose (Figure S5 in the Supporting Information).

**Figure 5:**
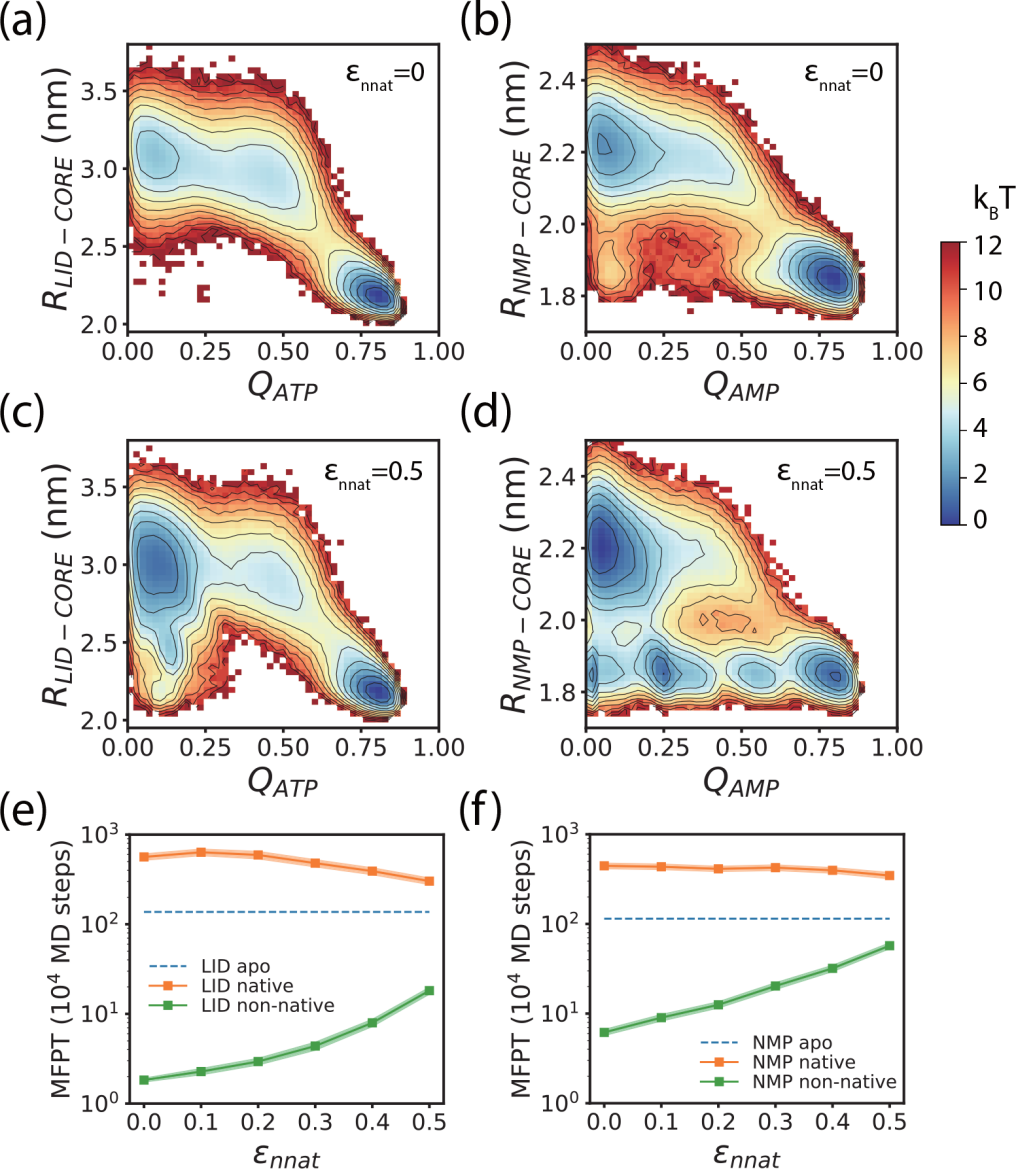
Thermodynamic and kinetic properties of the enzyme–substrate system in the coarse-grained simulations based on the WT model. (a and b) Two-dimensional free energy profiles along the reaction coordinates *R*_*LID*–*CORE*_ and *Q*_*ATP*_ (a) and along the reaction coordinates *R*_*NMP*–*CORE*_ and *Q*_*AMP*_ (b) with ε_*nnat*_ = 0. (c and d) Same as a and b but with ε_*nnat*_ = 0.5. (e) MFPTs of the LID domain opening with native substrate binding pose (orange) and non-native substrate binding pose (green) as a function of the strength of non-native contacting interactions ε_*nnat*_. For comparison, results at the apo state (without substrate binding) are also shown (blue dashed line). (f) Same as e but for the MFPTs of the NMP domain opening. Error bars were computed by the bootstrap method.

The observed effect of energy landscape ruggedness on substrate binding can also be illustrated by the free energy landscapes along the reaction coordinates *R*_*LID*–*CORE*_ (*R*_*NMP*–*CORE*_) and *Q*_*ATP*_ (*Q*_*AMP*_). For the case of ε_*nnat*_ = 0, the free energy landscapes are smooth and have two major basins, including the closed conformation with a native substrate binding pose and the open conformation with partial binding of substrate molecules (small *Q* values) (Figure 5a and 5b). For the case of larger ε_*nnat*_, the non-native interactions tend to cause additional substates along the free energy profile, which are featured by non-native substrate binding poses (low *Q* values) at the closed conformation of AdK (Figure 5c and 5d).

### Effect of Substrate Binding State on the Opening Rate of AdK Domains Probed with Coarse-Grained Simulations

Next, we investigated how the substrate binding state affects the domain opening rate in the coarse-grained simulations. We computed the MFPT of the conformational switching from the closed conformation to the open conformation for the LID and NMP domains with the substrate molecules being prebound with native binding poses and non-native binding poses, respectively. For comparison, we also calculated the MFPT for the apo enzyme. All of these simulations were performed for the WT model of the enzyme with different ε_*nnat*_ values.

As shown in Figure 5e and 5f, for both the LID domain and the NMP domain, the MFPT of domain opening is much longer when the substrates were prebound at the binding sites with native-like binding poses compared to that without substrate binding. These results suggest that correct substrate binding increases the stability of the closed conformation of the enzyme and therefore slows down domain opening, which is in line with the free energy landscape obtained based on all-atom umbrella sampling simulations (Figure 2c). However, when the substrate molecules were prebound around the binding site with non-native poses, the MFPT of domain opening became dramatically smaller compared to that without substrate binding. The free energy landscapes shown in Figure 5c and 5d also illustrate that when the substrates are bound incorrectly (e.g., with *Q*_*ATP*_(*Q*_*AMP*_) < 0.25), the free energy barrier becomes much smaller. For example, when the ATP is bound correctly, the free energy barrier for LID domain opening is ∼6*k*_*B*_*T* (Figure 5c), whereas the free energy barrier vanishes when the ATP adopts a fully non-native binding pose (Figure 5c). These results directly demonstrate that non-native substrate binding tends to speed up the domain opening, which is also consistent with the results from all-atom umbrella sampling simulations. With increasing values of ε_*nnat*_, the MFPT of domain opening with non-native binding poses increases. A possible reason for increased MFPT at higher ε_*nnat*_ values is that the substrate-mediated non-native interactions increase the ruggedness of the energy landscape of conformational motions and slow down domain opening.

## Discussion and Conclusion

How protein dynamics contribute to function is one of the current focuses in molecular biophysics. As a model system, AdK has been widely used to study the dynamics–function relationship of enzymes. In the enzymatic cycle of AdK, substrate binding and product release tend to occur at a relatively open conformation whereas the phosphoryl transfer reaction requires a catalytically competent closed conformation with the substrates and the residues at the active sites being precisely preorganized. Therefore, the conformational transition between a closed conformation and an open conformation is essential for the catalytic cycle of AdK. An early study showed that the intrinsic conformational fluctuations of the substrate-free AdK are not random but highly correlated to the conformational motions required for accessing the catalytically competent state.^6^ It has also been well established that such conformational motions can be modulated by ligand binding, as shown in many other allosteric proteins.^47,62−64^ However, the detailed molecular mechanism of how the conformational motions contribute to productive substrate binding is not very clear.

**Figure 6:**
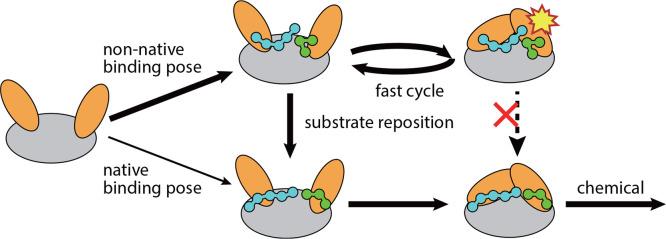
Schematic of the interplay between substrate binding and conformational dynamics. The encounter complex of substrate binding is dominated by non-native binding poses. Conformational closing of the enzyme with non-native binding poses results in a high-energy intermediate state (indicated by the yellow star), which cannot directly rearrange to the reactive native binding pose (red cross). Instead, the enzyme needs to perform repeated conformational transitions for the substrate to relax to the reactive native binding pose.

In this work, we addressed this question by performing multiscale MD simulations. The simulation results showed that the initial encounter complex may adopt heterogeneous conformations and the substrates dominantly bind with non-native poses (Figure 1c). The mobility of substrates in the substrate binding pocket depends on the conformational state of AdK. Starting from the non-native binding pose, the substrates are able to reposition at the open conformation of AdK (Figure 6), whereas in the closed conformation, the substrates have low mobility due to the steric confinement and thus are difficult to rearrange to the native binding pose. Consequently, successful substrate binding events are more likely when AdK repeatedly opens and closes as shown by both the all-atom MD simulations and the coarse-grained MD simulations. In addition, when the substrate has relaxed to a near-native pose, the domain closing action may further push it to the correct binding structure (Figure 4). These results directly demonstrate that the opening and closing motions of AdK help the substrates to find the native binding pose. The free energy calculations based on umbrella sampling simulations also showed that when the substrates bind with a non-native pose, the free energy profile of domain opening motions may become downhill-like, lacking a high free energy barrier. Therefore, the time scale for domain opening becomes extremely fast, which may explain the experimentally observed ultrafast conformational dynamics under turnover conditions.^31^ On the other hand, when the substrates successfully bind to the binding pockets with a native pose, the closed conformation becomes much stabilized, which may favor the subsequent chemical reaction step. Our results are in line with a previous proposition that specific interactions of the substrate with the residues involved in the catalytic reaction are also responsible for the change in conformational dynamics.^31^

Recent experimental works suggested a two-step mechanism of substrate binding for the AdK enzymatic cycle.^65,66^ In the first step, substrates bind to AdK at the partially open conformation to form a high-energy intermediate state. In the second step, the high-energy state converts to the fully closed conformation by rearrangement of the residues of the binding sites through an induced fit mechanism. The non-native binding and rearrangement mechanism revealed in this work is in line with the above two-step mechanism. However, we showed that repeated conformational transitions are necessary for the second step, particularly for the rearrangement of the substrate molecules. The results in this work also suggested that the substrate binding of AdK involves switching between different free energy profiles (FEPs). In addition to the FEPs at the unbound state and the natively bound state, other FEPs with non-native binding poses also contribute to the overall dynamics of substrate binding. Our findings provide mechanistic insights into previous experimental observations and augment our understanding of the general principles utilized by natural enzymes to achieve enormous catalytic power.

## Supporting Information Available

Additional results of coarse-grained and all-atom MD simulations, including the trajectories and probabilities of contact formation (PDF)
