# Supplementary material for: Role of Repeated Conformational Transitions in Substrate Binding of Adenylate Kinase
Source: J Phys Chem B. 2022 Oct 12;126(41):8188–201. doi: 10.1021/acs.jpcb.2c05497 (PMC9589722; doi:10.1021/acs.jpcb.2c05497)
Supplement: Supplementary file 1 — jp2c05497_si_001.pdf [file jp2c05497_si_001.pdf]

Supporting Information

Role of Repeated Conformational Transitions in Substrate Binding of Adenylate Kinase

Jiajun Lu,[†,‡] David Scheerer,[¶] Gilad Haran,[*,¶] Wenfei Li,[*,†,‡] and Wei Wang[*,†]

†*Department of Physics, National Laboratory of Solid State Microstructure, Nanjing University, Nanjing 210093, China*

‡*Wenzhou Key Laboratory of Biophysics, Wenzhou Institute, University of Chinese Academy of Sciences, Wenzhou, Zhejiang 325000, China*

¶*Department of Chemical and Biological Physics, Weizmann Institute of Science, Rehovot 761001, Israel*

E-mail: gilad.haran@weizmann.ac.il; wfli@nju.edu.cn; wangwei@nju.edu.cn

Figure S1

Figure S2

Figure S3

Figure S4

Figure S5

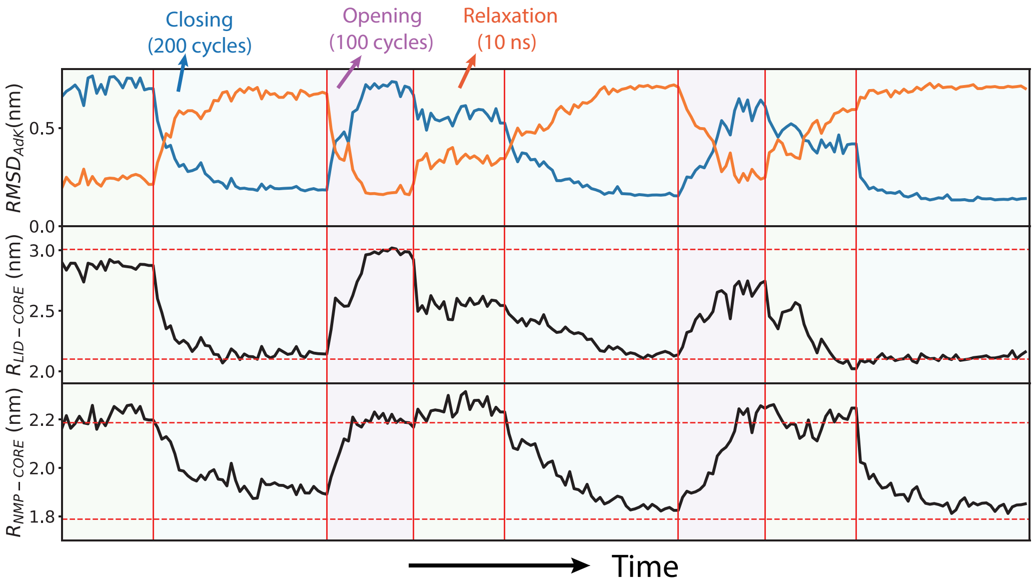

Figure S1: Representative PaCS-MD trajectory. (top) PaCS-MD trajectory shown by $RMSD_{AdK}$, which represents the root mean square deviations of the protein AdK with respect to the closed conformation (blue) or open conformation (orange); (middle) PaCS-MD trajectory shown by the distance $R_{LID-CORE}$ between the LID domain and CORE domain. (bottom) PaCS-MD trajectory shown by the distance $R_{NMP-CORE}$ between the NMP domain and CORE domain. The red dash lines represent the corresponding distances at the open and closed crystal structures. The distance between the centers of mass of the corresponding domains were used to represent the inter-domain distance. The results show that the inter-domain distances can vary in wide range, suggesting that the conformational transitions observed in the PaCS-MD simulations reflect the large-scale closed/open transitions measured in single-molecule experiment. Because the ATP is prebound with a native pose in the simulations, the LID domain can more easily access the fully closed conformation as shown in the middle panel. In comparison, it is difficult for the NMP domain to access the fully closed conformation before the substrate AMP finds the native binding pose.

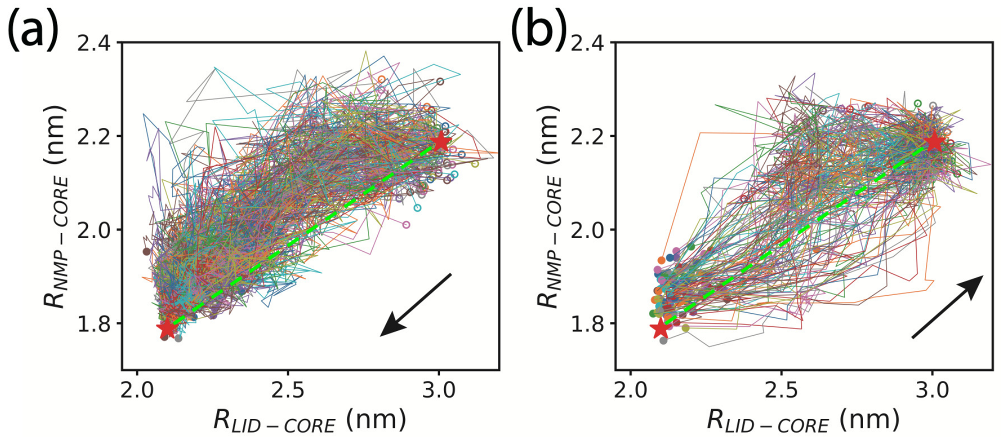

Figure S2: PaCS-MD trajectories plotted along the inter-domain distances $R_{LID-CORE}$ and $R_{NMP-CORE}$ for the open-to-closed transitions (a) and the closed-to-open transitions (b). Different colors represent different trajectories. The open circles and closed circles represent the two end structures of the transition events. The red stars correspond to the locations of the open and closed crystal structures, and the green dash lines are drawn to schematically indicate the most straightforward transition path between the two states. The black arrows represent the transition directions.

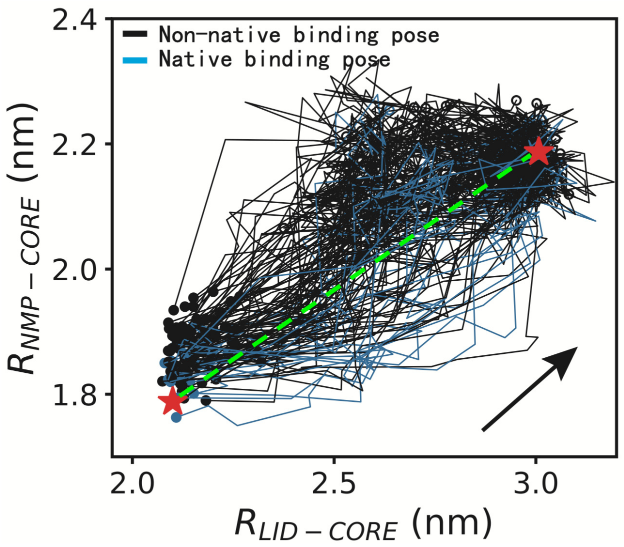

Figure S3: Same as Fig. S2b but with the trajectories drawn by a different color scheme. Blue: Trajectories for which the substrate AMP has a native-like binding pose at the beginning of the closed-to-open-transitions according to the criterion defined in Methods section; Black: all the other closed-to-open-transition trajectories. One can see that when the substrate AMP has already relaxed to the native-like binding pose at the beginning of the closed-to-open-transitions, the transition events dominantly initiate with the opening of the LID domain.

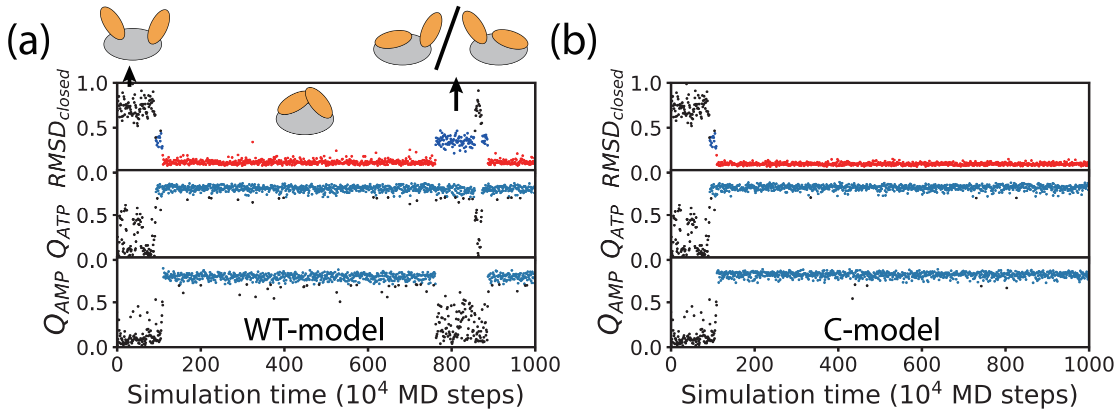

Figure S4: (a) Representative trajectory showing the time series of the root mean square deviation of the enzyme with respect to the closed conformation ($RMSD_{closed}$), the fraction of native contacts formed by ATP (Q_{ATP}) and AMP (Q_{AMP}) based on the WT-model and with $\varepsilon_{nnat} = 0$. In the $RMSD_{closed}$ trajectory (top), the red, blue, and black dots represent the fully closed conformation, one-domain closed conformation, and open conformation, respectively. In the Q_{ATP} and Q_{AMP} trajectories (middle and bottom), the snapshots with successful substrate binding were marked light blue. (d) Representative trajectory based on the C-model and with $\varepsilon_{nnat} = 0$.

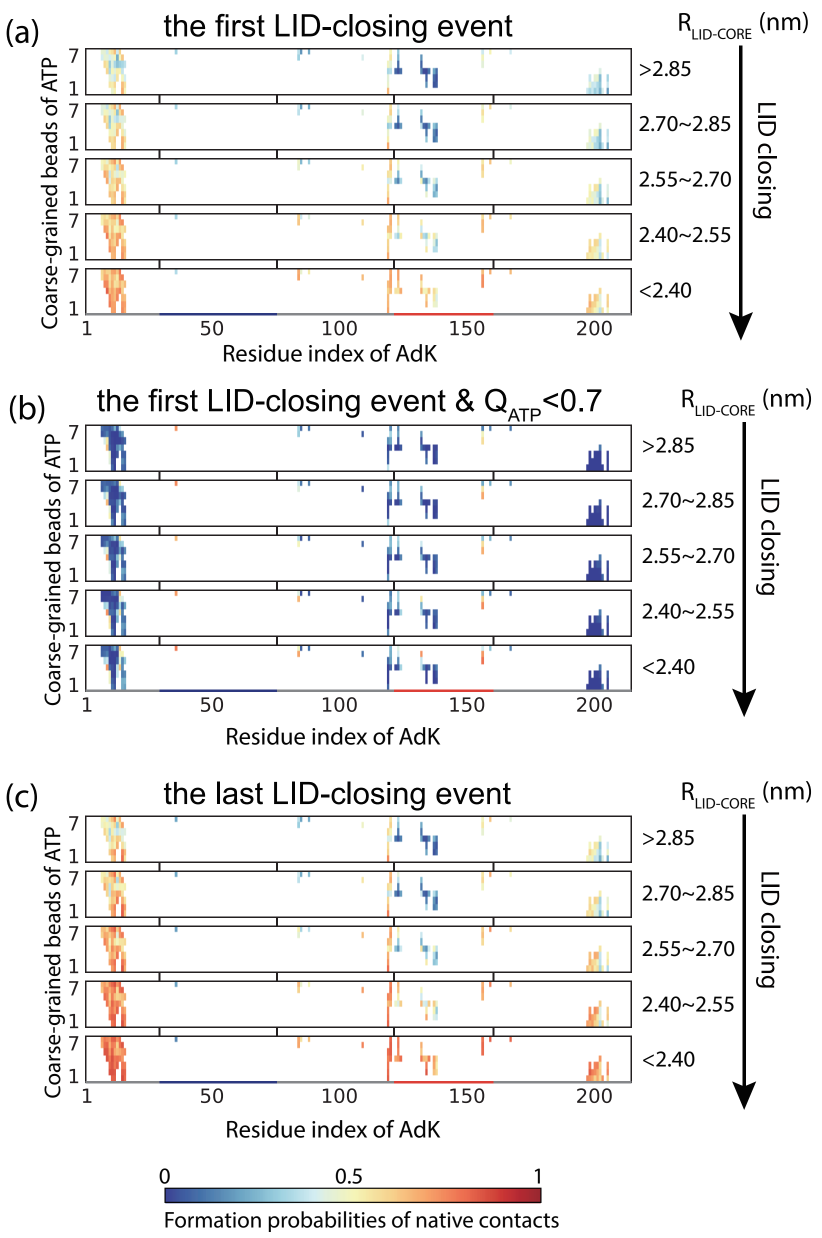

Figure S5: (a)Probabilities of formed native contacts between coarse-grained beads of the substrate ATP and the protein AdK at different stages of the first open-to-closed transition event of a complete substrate binding trajectory leading to the correct substrate binding state. The results were calculated based on 96 independent simulation trajectories with $\varepsilon_{nnat} = 0.5$. For a better comparison, the trajectories with the substrates staying out of the binding area during the conformational closing events were excluded. (b) Same as (a) but only for the first open-to-closed transition events with the ATP bound incorrectly ($Q_{ATP} < 0.7$). We note that the first LID domain closing events mostly occur when the ATP adopts a near-native binding pose. Therefore, the LID domain closing events with the ATP bound incorrectly are relatively rare. (c) Probabilities of formed native contacts between coarse-grained beads of substrate ATP and protein AdK at different stages of the last open-to-closed transition events.
